# *Guibourtia tessmannii*-induced fictive ejaculation in spinal male rat: involvement of D_1_, D_2_-like receptors

**DOI:** 10.1080/13880209.2017.1291692

**Published:** 2017-02-19

**Authors:** Patrick Brice Deeh Defo, Elvis Asongu, Modeste Nya Wankeu, Esther Ngadjui, Georges Romeo Bonsou Fazin, François Xavier Kemka, Miguel Carro-Juarez, Albert Kamanyi, Pierre Kamtchouing, Pierre Watcho

**Affiliations:** a Animal Physiology and Phytopharmacology Laboratory, University of Dschang, Dschang, Cameroon;; b Department of Animal Organisms Biology, University of Douala, Douala, Cameroon;; c Laboratorio de Comportamiento Reproductivo, Escuela de Medicina Veterinaria y Zootecnia, Universidad Autonoma de Tlaxcala-Mexico, Tlaxcala, Mexico;; d Department of Animal Biology and Physiology, University of Yaoundé, Yaoundé, Cameroon

**Keywords:** *Guibourtia tessmannii*, spinal cord, fictive ejaculation, D1-D2 receptors

## Abstract

**Context:***Guibourtia tessmannii* (Caesalpiniaceae) is a plant traditionally used as aphrodisiac. We previously reported the pro-ejaculatory effects of the aqueous and methanol extracts of *G. tesmannii* in spinal male rat. However, the mechanism underlying such effects has not been elucidated.

**Objective:** This study characterizes the dopaminergic sub-type receptors involved in *G. tesmannii*-induced ejaculation in male Wistar rat.

**Materials and methods:** Urethane-anesthetized spinal male rats were intravenously treated with saline solution (1 mL/kg, control); dopamine (0.1 μmol/kg, reference); aqueous or methanol extracts of *G. tesmannii* (20 mg/kg) in the absence or presence of haloperidol (0.26 μmol/kg), a nonspecific dopaminergic receptor antagonist, Sch23390 (0.26 μmol/kg), a specific D1-like receptor antagonist or, sulpiride (0.26 μmol/kg), a specific D2-like receptor antagonist. Electromyography of the bulbospongiosus muscles and intraseminal pressure were recorded after urethral, penile and drug stimulations.

**Results:** Urethral and penile stimulations, intravenous injection of dopamine or, aqueous and methanol extracts of *G. tesmannii* always triggered the expression of rhythmic contraction of the bulbospongiosus muscles with an average mean of 3.33 ± 0.43; 7.83 ± 0.85; 9.80 ± 0.86; 0.83 ± 0.54 and 2.67 ± 0.95 contractions, respectively. The intraseminal pressure was more expressed after urethral and penile stimulations (15.66 ± 1.58 and 13.60 ± 2.40 mmHg, respectively). In rats pretreated with haloperidol, Sch23390 or sulpiride, no ejaculation was recorded after intravenous injection of *G. tesmannii* extracts or dopamine.

**Discussion and conclusion:***Guibourtia tesmannii-*induced ejaculation requires the integrity of D_1_ and D_2_-like receptors. These findings further justify the ethno-medicinal claims of *G. tesmannii* as an aphrodisiac.

## Introduction

Ejaculation is considered as the physiological process that describes the expulsion of semen from the urethra and comprises two successive phases, emission and expulsion that require the participation of different pelvi-perineal anatomical structures (Truitt & Coolen 2002; Veening & Coolen 2014). The emission phase consists of secretion of the various components of semen by seminal vesicles, prostate and through the release of ampullary vasa deferentia contents into the prostatic urethra. The expulsion phase corresponds to the forceful propulsion of sperm from the prostatic urethra to the urethral meatus through rhythmic contractions of perineal striated muscles, which is the primary role of the bulbospongiosus muscles (Sheu et al. 2014). Ejaculation, which is basically a spinal reflex, requires a tight coordination between sympathetic, parasympathetic, and somatic efferent pathways originating from different segments and area in the spinal cord and innervating pelvi-perineal anatomical structures. The spinal ejaculation generator triggers ejaculation by integrating sensory inputs conveyed by the dorsal penile nerve, the sensory branch of the pudendal nerve, during copulation with autonomic and motor outflow (Staudt et al. 2011; Staudt et al. 2011, 2012). Neurotransmitters such as dopamine and serotonin are involved in the control of ejaculation (Sanna et al. 2014; Cooper et al. 2015).

Dopamine is the main neurotransmitter that plays a central role in the control of ejaculation (Giuliano 2011; Sanna et al. 2014). Mammalian dopamine receptors have been fully characterized and classified into D1-like receptors (D1 and D5) and D2-like receptors (D2, D3 and D4). Activation of dopaminergic receptors by systemic dopamine (Watcho & Carro-Juarez 2009) or apomorphine (Zaringhalam et al. 2010), a nonselective dopamine receptor agonist triggers the ejaculatory process in spinal male rats. Systemic administration of antagonists of dopaminergic receptors abolishes the expression of the ejaculatory response in spinal rats (Clément et al. 2009; Watcho et al. 2013). Haloperidol (a nonspecific dopamine antagonist), Sch23390 (a D1-like dopamine antagonist) and sulpiride (a D2-like dopamine antagonist) were used in the present study.

Plants are valuable resources in the health system of developing countries. Although there are no accurate data to assess the extent of global use of medicinal plants, the World Health Organization (WHO) has estimated that over 80% of the world's population uses traditional medicine routinely to meet their primary health care (Ortega et al. 2006), and that much of the traditional treatments involve the use of plant extracts or their active principles (Akerele 1985). Traditional herbs have importantly contributed to revolutionary breakthrough in the management of sexual inadequacies and specifically, in the context of ejaculatory physiology (Adimoelja 2000). For instance, *Bersama engleriana* (Melianthaceae) and *Dracaena arborea* (Dracaenaceae) extracts prevent the proejaculatory effect of dopamine (Watcho & Carro-Juarez 2009; Watcho et al. 2014) whereas cihuapatli (*Montanoa* genus) increase the ejaculatory potency in spinal male rats (Carro-Juarez et al. 2014).

In a preliminary study, we evaluated the pro-ejaculatory effects of the aqueous and methanol extracts of *Guibourtia tessmannii* (Caesalpiniaceae) in spinal male rats with the dose 20 mg/kg being the most efficient (Watcho et al. 2013). However, the mechanism of action of this plant is still unknown. The objective of this work was therefore to characterize the dopamine sub-type receptors (D1 and D2-like receptor) involved in the pro-ejaculatory activities of *G. tessmannii*. We employed the fictive ejaculation model in spinally and urethane anesthetized male rats (Watcho & Carro-Juarez 2009; Birri et al. 2014). This model permits the recording and visualization of the rhythmic motor pattern of ejaculation accompanied by complex pelvic activity that includes phasic and strong penile erections, as well as penile movements followed by the potent expulsion of urethral contents. The rhythmic motor pattern of ejaculation registered in this experimental model is elicited by urethral and penile stimulations and can additionally be induced by systemic administration of several drugs including medicinal plant extracts (Watcho et al. 2013; Carro-Juarez et al. 2014).

## Materials and methods

### Collection of plant material

The stem barks of *G. tessmannii* were collected in Ngoumou, a locality in the Mefou-Akono Division, Center Region of Cameroon in February 2015. Botanical identification was done by Dr. Victor Nana in the Cameroon National Herbarium (HNC) in comparison with the existing Herbarium Voucher specimen 1037/SRFCA. The stem bark was shade-dried and powdered using an electric grinder (Moulinex) and used to prepare aqueous and methanol extracts.

### Preparation of extracts

#### Aqueous extract

The powder from the stem barks of *G. tessmannii* (250 g) were macerated in 1.5 L of distilled water for 72 h. The macerate was filtered and the filtrate was oven dried (55 °C). The resulting material weighed 37.6 g with a percentage yield of 15.04% (w/w based on the dried starting weight).

#### Methanol extract

The powder of *G. tessmannii* (300 g) were macerated in 1.5 L of methanol for 72 h to yield, after solvent evaporation under reduced pressure, 34.1 g of brownish extract corresponding to an extraction yield of 11.37% (w/w based on the dried starting weight).

### Animals

Sexually experienced adult Wistar rats (> 90 days, 200–300 g body weight) were obtained from the animal house of the Department of Animal Biology of the University of Dschang, Cameroon. They were housed in groups, under natural light and day cycle and with free access to food and water. The experiments were done with respect to the internationally accepted standard of ethical guidelines for laboratory animal use and care as described in the European Community guidelines (EEC 1986). All efforts were made to minimize the number of rats used and their suffering.

### Sexual training

To provide sexual experience, each male rat was submitted to five consecutive sexual behavioral tests with an ovariectomized female brought to heat (estrus) experimentally with a single subcutaneous dose of 30 μg estradiol benzoate and 600 μg progesterone, 48 h and 6 h, respectively, before testing. In ovariectomized rat, it was shown that estradiol benzoate induced a specific urge to seek contact with a sexual active male (Meyerson & Lindstrom 1973). Behavioral observations were conducted after the onset of darkness and males were individually introduced into a cylindrical observation cage and an adaptation period (5–10 min) was allowed. Then, a receptive stimulus female was introduced and the copulatory behavior was permitted during a period of 25 min. Male rats exhibiting active sexual behavior and ejaculation latencies of less than 15 min in the last three sessions were used to classify them as sexually experienced male rats (Estrada-Reyes et al. 2009).

### Drugs

Urethane, dopamine, estradiol, progesterone (Sigma Chemicals, St. Louis, MO), haloperidol, sulpiride and Sch23390 (MP Biomedicals, Inc, Germany) were used in this study. Estradiol and progesterone were dissolved in ethanol and administered in oil while other chemicals were freshly prepared in saline solution. The doses were selected and used based on previous studies (Watcho et al. 2007; Watcho & Carro-Juarez 2009).

### Surgical process and, activation and recording of the rhythmic genital motor pattern of ejaculation

Rats were anesthetized with urethane (1.5 g/kg, ip), and by performing a surgical incision on the perineum, the bulbospongiosus genital muscles were identified and exposed. Two electrodes (EL 452, 12 mm, BIOPAC) were inserted into the bulbospongiosus muscles to record electromyograms (EMG). A catheter connected to a pressure transducer (SS13L, BIOPAC) was introduced into the seminal vesicles to record pressure. For a better visualization of the motor genital activity associated with the ejaculation, an additional surgery was performed to expose the bulbar portion of the penis and its anatomical connections with the striated bulbospongiosus muscles. At the end of the surgical approach, the spinal cord was blunt transected around T6 spinal level. Treatments were administered by infusing the selected drugs into the jugular vein.

After spinal cord transection, ejaculatory motor patterns was reflexively expressed and recorded in the genital muscles of all animals. To establish the capacity of the spinal apparatus to produce the genital rhythmic pattern after spinalization, two consecutive ejaculatory motor patterns were repeatedly evoked at 3 min intervals by the injection of saline solution (200 μl/min) through a PE-50 catheter (0.965 mm o.d.) inserted into the pelvic urethra through a bladder incision or by tactile stimulation of the penis. Injection of saline solution was directed to increase the intra-urethral pressure to simulate the urethral distension produced by the emptying of the contents of the accessory glands into the posterior urethra. Tactile stimulation of the penis was performed by creating a tonic pressure in the penis using two forceps. The first forceps was used to block the preputial sheath on the top of the penis while the arms of the second forceps were repetitively (4 to 5 times) and gently applied from the basis of the penis to the top (nearby the blocked preputial sheat area) in order to create pressure. Afterward, the two forceps were removed and spontaneous erections of the penis as well as contractions of the striated bulbospongiosus muscles could be observed and recorded. Thereafter, one of the selected treatments was intravenously applied and EMG of the bulbospongiosus muscles as well as intraseminal pressure was recorded for 5 min, by registering on a polygraph (Biopac Student Lab PRO, version 3.7.3, frequency 50 Hz and model MP36E-CE). Five minutes after recording the EMG and intraseminal pressure in each sequential treatment, three consecutive urethral stimulations were monitored at 3 min intervals, as described above. The latency of contractions was expressed as the time elapsed from the application of a test stimulus until the first contraction of the bulbospongiosus muscles. The number of motor contractions included all motor contractions expressed in the motor ejaculatory train evoked by the sensory or pharmacological stimuli. The frequency of contractions of the bulbospongiosus muscles was calculated by dividing the number of contractions by the duration of the motor train (Carro-Juarez & Rodrıguez-Manzo 2000; Watcho & Carro-Juarez 2009; Watcho et al. 2013, 2014).

### Experimental treatment

Animals were randomly divided into 13 groups of five rats each and intravenously treated with one of the following: saline solution (1 mL/kg, control); dopamine (0.1 μmol/kg); aqueous extract of *G. tessmannii* (20 mg/kg); methanol extract of *G. tessmannii* (20 mg/kg); haloperidol (0.26 μmol/kg) plus aqueous extract of *G. tessmannii* (20 mg/kg); haloperidol (0.26 μmol/kg) plus methanol extract of *G. tessmannii* (20 mg/kg); sulpiride (0.26 μmol/kg) plus aqueous extract of *G. tessmannii* (20 mg/kg); sulpiride (0.26 μmol/kg) plus methanol extract of *G. tessmannii* (20 mg/kg); SCH23390 (0.26 μmol/kg) plus aqueous extract of *G. tessmannii* (20 mg/kg); SCH23390 (0.26 μmol/kg) plus methanol extract of *G. tessmannii* (20 mg/kg); haloperidol (0.26 μmol/kg) plus dopamine (0.1 μmol/kg); sulpiride (0.26 μmol/kg) plus dopamine (0.1 μmol/kg); SCH23390 (0.26 μmol/kg) plus dopamine (0.1 μmol/kg). The doses used in this study were selected based on our pilot/previous study (Watcho et al. 2013).

In all combined treatments, the second drug was injected 30s after the first one. The products were administered intravenously with a volume of administration of 0.2 mL/rat for each drug and an infusion time of 5s.

### Statistical analysis

All results were expressed as the mean plus or minus standard error of mean (M ± SEM). The statistical tests were performed with Stat Soft, Inc. (2008). STATISTICA (data analyzes software system), version 8.0.www.statsoft.com. Statistical significance was determined by one-way analysis of variance (ANOVA I) followed with *post hoc* Tukey HSD test for multiple comparisons. *p* Values <0.05 were considered statistically significant.

## Results

### Effects of urethral and penile stimulations and, intravenous administration of plant extracts on fictive ejaculation in spinal rat

**Figure 1.**
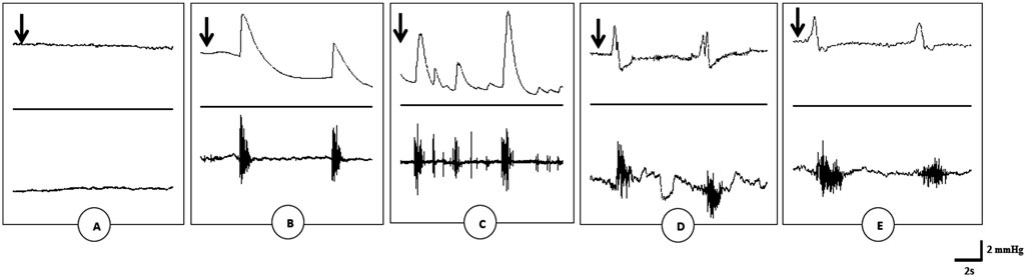
Polygraphic intraseminal pressure (upper) and EMG (lower) tracings showing the effects of saline solution (A), urethral stimulation (B), penile stimulation (C), aqueous extract (D) and methanol extract of *G. tessmannii* (20 mg/kg) (E) in spinal male rats. Arrows indicate the moment of substance application.

Intravenous injection of saline solution (2 mL) failed to trigger the rhythmic contraction of the striated bulbospongiosus muscles (Figure 1A; Table 1). On the contrary, injection of saline solution into the urethra via the bladder (urethral stimulation), penile stimulation, aqueous and methanol extracts of *G. tessmannii* (20 mg/kg) provoked rapid rhythmic contractions of the bulbospongiosus muscles with an average mean of 3.33 ± 0.43; 7.83 ± 0.85; 0.83 ± 0.54 and 2.67 ± 0.95 contractions, respectively (Figure 1(B–E) and Table 1). These ejaculatory motor responses were sometimes followed by penile movements, erection and emission of the seminal plugs. It is also noteworthy mentioning that all the time, the activities of the muscles involved in the expulsion phase of ejaculation were coupled to an expression of the intraseminal pressure (emissive phase of ejaculation) with an average mean of 15.66 ± 1.58; 13.60 ± 2.40; 6.45 ± 0.62; 2.20 ± 0.29 and 7.08 ± 2.31 mmHg for urethral stimulation, penile stimulation, dopamine (0.1 μmol/kg) and, aqueous and methanol extracts of *G. tessmannii* (20 mg/kg), respectively (Figure 1; Table 1).

**Table 1.** Effects of penile and urethral stimulations, intravenous administration of drugs on the number, frequency and latency of contractions of the bulbospongiosus muscles in spinal rats.

Treatments	Intraseminal pressure (mm Hg)	Number of contractions (N)	Frequency of contractions (N/s)	Latency of Contractions (s)	Ejaculatory motor response
Penile stimulation	13.60 ± 2.40 2.40122.4012.401	7.83 ± 0.85	0.38 ± 0.02	2.75 ± 0.47	+
Urethral stimulation	15.66 ± 1.58	3.33 ± 0.43	0.44 ± 0.04	9.50 ± 1.69	+
Dopamine (0.1 μmol/kg)	6.45 ± 0.62	9.80 ± 0.86^###^	0.41 ± 0.02	14.60 ± 3.34^### ФФФ^	+
Aqueous extract of *G. tesmannii* (20 mg/kg)	2.20 ± 0.29 ^##ФФФ^	0.83 ± 0.54^ФФ^***	0.22 ± 0.16	ND	+
Methanol extract of *G. tesmannii* (20 mg/kg)	7.08 ± 2.31^ФФ^	2.67 ± 0.95^Ф^***	0.68 ± 0.34	463.40 ± 81.78^### ФФФ***^	+
Haloperidol (0.26 μmol/kg) + aqueous extract of *G. tesmannii* (20 mg/kg)	0	0	0	ND	−
Haloperidol (0.26 μmol/kg) + methanol extract of *G. tesmannii* (20 mg/kg)	0	0	0	ND	−
Sch23390 (0.26 μmol/kg) + aqueous extract of *G. tesmannii* (20 mg/kg)	0	0	0	ND	−
Sch23390 (0.26 μmol/kg) + methanol extract of *G. tesmannii* (20 mg/kg)	0	0	0	ND	−
Sulpiride (0.26 μmol/kg) + aqueous extract of *G. tesmannii* (20 mg/kg)	0	0	0	ND	−
Sulpiride (0.26 μmol/kg) + methanol extract of *G. tesmannii* (20 mg/kg)	0	0	0	ND	−
Haloperidol (0.26 μmol/kg) + Dopamine (0.1μmol/kg)	0	0	0	ND	−
Sulpiride (0.26 μmol/kg) + Dopamine (0.1μmol/kg)	0	0	0	ND	−
Sch23390 (0.26 μmol/kg) + Dopamine (0.1μmol/kg)	0	0	0	ND	−

Number of rats per group =5. All values are expressed as mean ± SEM. Urethral and penile stimulations represent the mean value of all urethral and penile stimulations carried out in this study. For each rat, the frequency of contractions of the bulbospongiosus muscles was calculated by dividing the number of contractions by the duration of the motor train and the latency was observed during EMG recording. ND = not determined. + = present; − = absent. ###*p* < 0.001 significantly different compared with urethral stimulation.

Ф *p* < 0.05.

ФФ *p* < 0.01.

ФФФ *p* < 0.001 significantly different compared with penile stimulation.

* *p* < 0.05.

** *p* < 0.01.

*** *p* < 0.001 significantly different compared with dopamine.

Results of the study also revealed that the methanol extract was more efficient than the aqueous extract although its activity was significantly reduced compared to the one evoked by dopamine (Table 1).

### Effects of a pretreatment with haloperidol, sulpiride and Sch23390 on dopamine and *G. tessmannii*-induced fictive ejaculation

**Figure 2.**
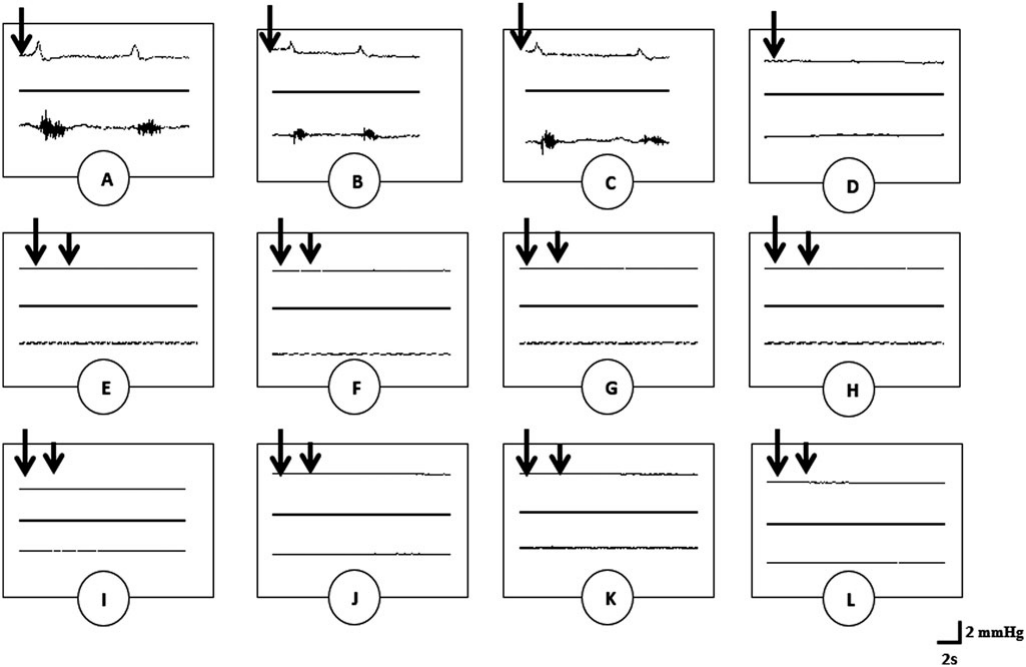
Polygraphic intraseminal pressure (upper) and EMG (lower) tracings showing the effects of dopamine (0.1 μmol/kg) (A), aqueous extract (B) and methanol extract (C) of *G. tessmannii* (20 mg/kg), haloperidol (0.26 μmol/kg) plus aqueous extract of *G. tessmannii* (20 mg/kg) (D), sulpiride (0.26 μmol/kg) plus aqueous extract of *G. tessmannii* (20 mg/kg) (E), Sch23390 (0.26 μmol/kg) plus aqueous extract of *G. tessmannii* (20 mg/kg) (F), haloperidol (0.26 μmol/kg) plus methanol extract of *G. tessmannii* (20 mg/kg) (G), sulpiride (0.26 μmol/kg) plus methanol extract of *G. tessmannii* (20 mg/kg) (H), Sch23390 (0.26 μmol/kg) plus methanol extract of *G. tessmannii* (20 mg/kg) (I), haloperidol (0.26 μmol/kg) plus dopamine (0.1 μmol/kg) (J), sulpiride (0.26 μmol/kg) plus dopamine (0.1 μmol/kg) (K), Sch23390 (0.26 μmol/kg) plus dopamine (0.1 μmol/kg) (L) on the bulbospongiosus muscles and seminal vesicles, respectively. Long and short arrows indicate the first and second injections, respectively.

It was clearly shown that intravenous injection of dopamine (0.1 μmol/kg) as well as plant extracts via the jugular vein always induced contractions of the striated bulbospongiosus muscles and evoked seminal pressure (Figure 2(A–C) and Table 1).

Pre-treatment of spinal cord transected and urethane-anesthetized rats with haloperidol (0.26 μmol/kg) a nonspecific dopaminergic antagonist, Sch23390 (0.26 μmol/kg) a D1-like dopaminergic receptors antagonist or sulpiride (0.26 μmol/kg) a D2-like dopaminergic receptors antagonist 30 s before intravenous injection of aqueous or methanol extract of *G. tessmannii* (20 mg/kg) completely prevented the rhythmic contractions of the bulbospongiosus muscles and expression of the seminal pressure (Figure 2(D–I) and Table 1). Similar results were observed in the combined treatments of these inhibitors with dopamine (Figure 2(J–L) and Table 1).

## Discussion

Ejaculation is a complex sexual response which consists of seminal emission (secretion of the mixed fluids composing semen into the posterior urethra), expulsion (propulsion of semen from the posterior urethra to the outside) and bladder neck closure (Truitt & Coolen 2002; Veening & Coolen 2014). In a normal condition, these events occur reflexively, and require coordination of autonomic and somatic nervous system in order to achieve effective delivery of semen (Grillner 1985; Giuliano & Clement 2005; Yonezawa et al. 2009; Gajjala & Khalidi 2014; Huang et al. 2016; Kozyrev et al. 2016). In our previous work on the pro-ejaculatory effect of *G. tessmannii* in spinal male rats, no mechanism of action was suggested (Watcho et al. 2013). In order to clarify this issue, the main objective of this study was to characterize the dopaminergic sub-type receptors involved in the pro-ejaculatory activities of *G. tessmannii* in male rat.

Thus, we used again the fictive ejaculation model in spinally and urethane anesthetized male rats, which permits the recording of the rhythmic motor pattern of ejaculation that can be induced by sensory and pharmacological means (Watcho & Carro-Juarez 2009; Birri et al. 2014; Watcho et al. 2014). It has been reported that spinal cord transection exerts a facilitatory effect on the expression of sexual reflexes (Kozyrev et al. 2016). Further, experiments with acutely and chronically spinalized rats have shown that some rhythmic motor patterns can be evoked in these animals, evidencing that the spinal cord itself contains neural circuits that can coordinate the different muscles to produce locomotor movements or rhythmic patterns (Grillner 1985; Birri et al. 2014). Pressure in the seminal vesicles and contraction of the bulbospongiosus muscles are regarded as physiological markers of respectively, emission and expulsion phases of ejaculation (Giuliano & Clement 2005).

In the present study, urethral and penile stimulations always induced fictive ejaculation, characterized by rapid rhythmic contractions of bulbospongiosus muscles. It has been shown that in the fictive ejaculation model, the number of genital motor patterns of ejaculation evoked by urethral or penile stimulations is related to the ejaculatory capacity of male rats (Carro-Juarez et al. 2009). The expression of the genital motor patterns of ejaculation can be induced by repeated stimulation of the urethra. Once the supra-spinal inhibitory influences are removed by spinal cord transection, a spinal intrinsic rhythm of activation of the motor pattern of ejaculation is turned on (Giuliano & Clement 2005; Birri et al. 2014) and the urethral stimulation activates a sensory feedback mechanism that exerts both facilitatory and inhibitory influences on the ejaculatory trains (Carro-Juarez & Rodrıguez-Manzo 2005).

In all spinal rats, the intravenous injection of dopamine always induced fictive ejaculation. Dopamine is a neurotransmitter that facilitates ejaculatory responses at both central and peripheral levels (Kitrey et al. 2007; Giuliano, 2011; Domínguez-Salazar et al. 2014). Similar to dopamine, the intravenous injection of aqueous or methanol extracts of *G. tessmannii* (20 mg/kg) triggered rapid rhythmic contractions of the bulbospongiosus muscles which were also coupled to the expression of the seminal pressure. However, the number of discharge in the genital motor pattern of ejaculation was less compared to dopamine. These pro-ejaculatory effects of *G. tessmannii*, which confirmed the previous ones (Watcho et al. 2013) could once more be attributed to the presence of saponins, alkaloids and phenolic compounds particularly in the methanol extract. Thus *G. tessmannii* extracts promote the expression of genital motor patterns of ejaculation which could justify its traditional used as an aphrodisiac. Similarly, the pro ejaculatory effects of *Montanoa frutescens* and *Montanoa grandiflora* (Asteraceae) have been published (Carro-Juarez et al. 2014).

Because many compounds exert their pro-ejaculatory activity mainly through the dopamine system, the effects of *G. tessmannii* on the dopamine receptors was targeted in this work. Hence, a pretreatment of rats with either a nonspecific dopamine receptor antagonist, haloperidol (0.26 μmol/kg), a D1 dopamine receptor antagonist, Sch23390 (0.26 μmol/kg) or a D2 dopamine receptor antagonist, sulpiride (0.26 μmol/kg) prevented the rhythmic contraction of the bulbospongiosus muscles and the expression of seminal pressure due to *G. tessmannii*. The D1-like receptors stimulate adenylate cyclase and is made of D1 and D5 subtypes receptors while the dopamine D2-like which comprised of D2, D3 and D4 subtypes inhibits it (Cooper et al. 1986; Cadet et al. 2010). It has been shown that the stimulation of D2-like receptors is more efficient to facilitate fictive ejaculation in anesthetized rats (Clément et al. 2006). Present results strongly suggest that this pro-ejaculatory effect of *G. tessmannii* requires the integrity of D1 and D2-like receptors.

## Conclusion

With no doubt, the pro-ejaculatory effect of *G. tessmannii* is mediated via D1, D2-like dopamine receptors. These findings further justify the ethno-medicinal claims of *G. tessmannii* as an aphrodisiac. However, further investigation of the effects of *G. tessmannii* in animals with real ejaculatory dysfunction such as obese rats is highly needed.
